# Atypical neural modulation in the right prefrontal cortex during an inhibitory task with eye gaze in autism spectrum disorder as revealed by functional near-infrared spectroscopy

**DOI:** 10.1117/1.NPh.5.3.035008

**Published:** 2018-09-05

**Authors:** Takahiro Ikeda, Masahiro Hirai, Takeshi Sakurada, Yukifumi Monden, Tatsuya Tokuda, Masako Nagashima, Hideo Shimoizumi, Ippeita Dan, Takanori Yamagata

**Affiliations:** a Jichi Medical University, Department of Pediatrics, Shimotsuke, Japan; b Jichi Medical University, Center for Development of Advanced Medical Technology, Shimotsuke, Japan; c University of London, Centre for Brain and Cognitive Development, London, United Kingdom; d International University of Health and Welfare, Department of Pediatrics, Nasushiobara, Japan; e Chuo University, Applied Cognitive Neuroscience Laboratory, Tokyo, Japan; f International University of Health and Welfare Rehabilitation Center, Nasu Institute for Developmental Disabilities, Otawara, Japan

**Keywords:** executive function, autism spectrum disorder, cortical hemodynamics, dorsolateral prefrontal cortex, optical topography, gaze direction

## Abstract

Autism spectrum disorder (ASD) is characterized by impairment in social communication and the presence of restricted and repetitive behaviors and interests. Executive function impairment is reportedly partially responsible for these symptoms. Executive function includes planning, flexibility, and inhibitory control. Although planning and flexibility in ASD have been consistently reported as atypical, the atypicality of inhibitory control remains controversial. As most previous studies have used nonsocial stimuli to investigate inhibitory control in ASD, the effects of socially relevant information on the inhibitory control system in individuals with ASD remain unclear. Therefore, we developed a go/no-go task with gaze stimuli and measured hemodynamic responses in the right prefrontal cortex (PFC), involved in inhibitory processing in both typically developing (TD) children and children with ASD, using functional near-infrared spectroscopy. Direct gaze induced commission errors to similar extents in both groups. Contrary to the behavioral responses, neural activation in the right PFC was modulated by gaze direction only in the TD group. These findings suggest that the gaze-processing mechanisms in the prefrontal region may be affected by atypical gaze processing in other brain regions during an inhibitory control task with socially relevant information in ASD.

## 1 Introduction

Autism spectrum disorder (ASD) is a complex neurodevelopmental disorder characterized by impairments in social interaction and communication accompanied by repetitive stereotyped behaviors and restricted interests.^1^ In addition, studies have shown atypicality of executive functions (EFs) in ASD.^2^^–^^4^

EFs are defined as functions regulating various cognitive processes necessary for goal-directed behavior, including planning, flexibility, working memory, attention, and inhibition.^3^^,^^5^ It should be noted that not all domains of EF are impaired in individuals with ASD, but rather selective impairment, such as in planning and flexibility, has been reported. In contrast, most studies have shown that the function of inhibitory control in ASD is largely preserved.^3^^,^^6^^–^^8^

The right prefrontal cortex (PFC) is a candidate brain region for the neural substrate underlying inhibitory control. The potential involvement of this region is supported by several neuropsychological and developmental studies.^9^^,^^10^ Findings in children with attention-deficit hyperactivity disorder (ADHD), who exhibit deficits mainly in inhibitory control, indicate that atypical hemodynamic responses may also underlie the altered inhibitory response in children with ASD.^11^^,^^12^ Most studies have shown a hypoactivation pattern in the right PFC region.^10^^,^^13^ In our previous studies, hemodynamic responses in the right PFC were robustly increased in typically developing (TD) children, but reduced in children with ADHD, during a go/no-go task, as revealed by functional near-infrared spectroscopy (fNIRS).^14^^–^^19^

Despite the growing evidence of atypical hemodynamic response patterns in the right PFC in children with ADHD during an inhibitory task, it remains unclear whether the neural responses related to inhibitory function are preserved in children with ASD. In a study of inhibitory control in adults with ASD, Schmitz et al.^20^ found hyperactivation in the bilateral inferior frontal areas during a go/no-go task. In contrast, two other studies have demonstrated that, compared to control groups, adults with ASD exhibit reduced hemodynamic responses in the right inferior frontal gyrus during go/no-go tasks.^21^^,^^22^ Moreover, differences in neural connectivity in the PFC have been reported between individuals with ASD and controls.^23^^,^^24^

Atypical patterns of hemodynamic responses in the PFC region have also been reported in ASD;^20^^–^^24^ however, these findings remain controversial. This controversy may be attributed to the different types of cognitive tasks used in functional neuroimaging. These studies have mainly adopted nonsocial artificial stimuli, such as geometric figures, alphabets, and animated characters, to reveal the neural responses in the PFC region.^20^^–^^26^ However, because the atypical processing of socially relevant information in individuals with ASD has been established, it is necessary to examine the function of inhibitory control in the context of socially natural experimental conditions. The PFC region is part of the social brain network, which processes socially relevant stimuli and tasks. For example, gaze direction could modulate dorsolateral PFC (DLPFC) activation when participants viewed faces with averted and direct gazes.^27^ Therefore, it is likely that socially relevant information can affect hemodynamic responses in the PFC during social tasks.

In addition, a functional magnetic resonance imaging (fMRI) study revealed differential cortical responses in the DLPFC induced by gaze direction in the TD and ASD groups. In this study, the participants were instructed to watch an animation of a walking man with his gaze directed toward or averted from them. The authors found that the patterns of neural responses in the DLPFC were enhanced in the TD group when the participants observed an averted gaze; however, the opposite pattern was observed in the ASD group.^28^

Therefore, in the present study, we aimed to examine the behavioral and hemodynamic correlations between inhibitory control and socially relevant visual stimuli in children with and without ASD. Because we had observed hypoactivation in the right PFC region in children with ADHD using fNIRS,^14^^–^^19^ we focused on the specific activation pattern in the right PFC during a go/no-go task with direct and averted gazes. We reasoned that if the function of inhibitory control was modulated atypically by socially relevant information in children with ASD, both their behavioral and hemodynamic responses in the right PFC would also be atypical compared to those in the TD group.

## 2 Methods

### 2.1 Participants

Twenty-two individuals with ASD (17 boys, mean age: 12.9 years, S.D.: 2.8 years) were recruited from Jichi Medical University (Shimotsuke, Tochigi, Japan) and the International University of Health and Welfare (Otawara, Tochigi, Japan). As a control group, 24 healthy TD children (12 boys, mean age: 13.6 years, S.D.: 2.2 years) participated in the study. The TD children were recruited from elementary, junior high, and high schools located near Jichi Medical University and the International University of Health and Welfare, and had no history of psychiatric or neurological problems. There was a marginally significant sex difference between the groups (p=0.07, Fisher’s exact test). The ages of the two groups were not significantly different [t (44)=0.96, p=0.34]. The intelligence quotient (IQ) scores of the participants were tested with the Japanese version of the Wechsler Intelligence Scale for Children III and IV (WISC-III and WISC-IV) (Japanese WISC-III Publication Committee, 1998; Japanese WISC-IV Publication Committee, 2010). The two editions contain the same material with minor differences, have continuity in their structures, and have shown strong correlations with full-scale IQ tests (r=0.89).^29^ The IQ scores of the participants with ASD (mean IQ: 97.9, S.D.: 14.6, range: 71 to 121) were significantly lower [t (44)=2.32, p=0.03] than those of the TD controls (mean IQ: 106.5, S.D.: 10.4, range: 91 to 126) (Table 1). All participants and their parents provided written informed consent before the experiment, which was conducted in conformity with the tenets of the Declaration of Helsinki and was approved by the Institutional Review Boards of Jichi Medical University and the International University of Health and Welfare.

**Table 1:** Demographic and clinical profiles for ASD and TD subjects.

Group	N (M/F)	Chronological age	IQ	AQ	PARS
		Mean (years)±SD	Mean±SD (range)	Mean±SD (range)	Mean±SD (range)
		Range (years; months)			
ASD	22 (17/5)	12.9±2.8 (8;4 to 17;8)	97.9±14.6 (71 to 121)	24.9±13.5 (10 to 43)	28.3±11.4 (11 to 54)
TD	24 (12/12)	13.6±2.20 (9;0 to 17;0)	106.5±10.4 (91 to 126)	10.6±5.5 (2 to 19)	1.75±2.7 (0 to 11)

Note: ASD, autism spectrum disorder; TD, typical development; M, male; F, female; IQ, intelligence quotient; AQ, autism-spectrum quotient; PARS, Pervasive Developmental Disorders Autism Society Japan Rating Scale.

### 2.2 Psychiatric Assessment

The diagnosis of the participants with ASD was established by trained pediatric neurologists (T.I., Y.M., M.N., H.S., and H.W.) based on the Diagnostic and Statistical Manual of Mental Disorders (DSM-5) criteria.^1^ The diagnosis was further confirmed by two questionnaires: the autism-spectrum quotient (AQ)^30^ test and the Pervasive Developmental Disorders Autism Society Japan Rating Scale (PARS).^31^ The AQ is an instrument used for evaluating autistic traits, with a scoring range from 0 to 50, that has been validated for the Japanese population.^32^ In this experiment, we used the AQ children version, and the mean score of the participants with ASD was 24.9 (S.D.: 13.5, range: 10 to 43), while that of the TD controls was 10.6 (S.D.: 5.5, range: 2 to 19). The PARS is a semistructured interview in Japanese assessing the severity of autistic symptoms, and its score correlates with that of the Autism Diagnostic Interview Revised (r=0.41).^31^ It includes 57 items describing autistic characteristics, with 34 of them representing behaviors observed during infancy, 33 during childhood, and 33 during adolescence and adulthood. In this study, 33 of the items on childhood or adolescence/adulthood behaviors were adopted. The mean PARS score of the participants with ASD was 28.3 (S.D.: 11.4, range: 11 to 54), and that of the TD controls was 1.8 (S.D.: 2.7, range: 0 to 11).

### 2.3 Stimuli and Apparatus

In the present study, we developed a new inhibitory task that combines the go/no-go task with socially relevant cues. In this task, a green or red dot was superimposed between the eyebrows of a female or male face, with a direct or averted gaze, generated from FaceGen^®^. The participants were seated facing a screen presenting these stimuli and asked to press a button only when a green (= go task), but not red (= no-go task), dot appeared. We used E-Prime^®^ (Psychology Software Tools, Pittsburgh, Pennsylvania) to create and present the stimuli and collect the behavioral responses.

### 2.4 Experimental Procedure

**Fig. 1:**
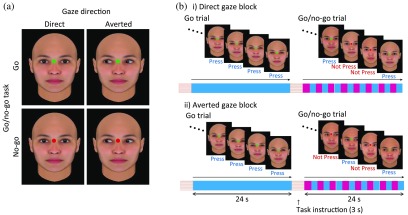
Experimental design and stimuli. (a) Direct or averted gazes with a green or red dot between the eyes were displayed as socially relevant visual stimuli. (b) Each block (direct or averted gaze condition) contained one go trial and one go/no-go trial. The duration of each trial was 24 s.

In this experiment, a block contained a go trial (in which only faces with closed eyes and a green dot between the eyebrows were shown) and a go/no-go trial (in which faces with either direct or averted gazes, and with either a green or red dot between the eyebrows, were shown). The participants underwent two sessions consisting of six blocks each, including three blocks for the direct gaze condition and three blocks for the averted gaze condition. Each trial in a block lasted for 24 s preceded by a 3-s instruction, “press the button for the green dot” or “do not press the button for the red dot.” The stimuli were displayed on the screen once per second. Therefore, in total, 24 tasks were displayed in a go/no-go trial (Fig. 1). The go/no-go trials included 50% of no-go tasks. The go/no-go ratio was determined based on previous behavioral and neuroimaging studies.^6^^,^^14^^–^^19^^,^^33^^–^^36^ To press the button, the participants had to focus their attention on the dots between the eyebrows. This ensured that the participants always observed the direction of the gaze during a trial. Because we displayed the face stimuli with closed eyes in the baseline period (the go trial), the neural responses specific for gaze direction could be isolated in the subsequent go/no-go trial. Gaze direction has been suggested to facilitate social cognitive function^37^^,^^38^ and induce neural responses in the PFC.^39^^,^^40^ Therefore, we sought to identify the effect of gaze stimuli on the inhibitory function in the PFC region.

### 2.5 fNIRS Measurements

**Fig. 2:**
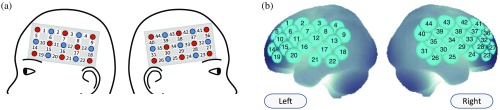
Spatial profiles of fNIRS channels. (a) The probe and channel locations on the scalp are shown in both left- and right-side views. The probes covered the bilateral frontal and temporal regions. The blue circles indicate detectors emitting infrared radiation, and the red circles indicate illuminators that receive reflected light. The numbers in the white squares between the blue and red circles indicate channel numbers. (b) Probabilistically estimated fNIRS channel locations on the brain surface (centers of blue circles) for all participants and their spatial variability (SD, radii of the blue circles) associated with the estimation are depicted in a Montreal Neurological Institute space.

fNIRS is a noninvasive neuroimaging tool for continuous measurement of cerebral cortical hemodynamics. Near-infrared light is irradiated at two wavelengths (695 and 830 nm), and signals are measured reflecting changes in the concentrations of oxygenated (oxy-Hb) and deoxygenated (deoxy-Hb) hemoglobin in the brain tissue at 25 to 30 mm from the surface, based on the modified Beer–Lambert Law.^41^ Since the oxy-Hb concentration increases and the deoxy-Hb concentration decreases in activated brain regions because of neurovascular coupling, changes in these parameters signify focal hemodynamic responses. Accordingly, we calculated signals reflecting oxy-Hb and deoxy-Hb concentration changes, expressed in units of millimolar·millimeter (mM·mm).^42^ We placed 44 channels over the bilateral PFC in the parietal scalp area to measure the oxy-Hb and deoxy-Hb signals in the frontal lobe, related to inhibitory control, with an ETG-4000^®^ multichannel fNIRS system (Hitachi Medical Corporation, Kashiwa, Japan)^14^^–^^19^ (Fig. 2).

For all participants, the position of each channel was obtained using a three-dimensional-digitizer (Patriot Digitizer^®^, Polhemus) after the fNIRS measurement, and the data were matched to the Montreal Neurological Institute (MNI) brain template form. This procedure is an established method of probabilistic registration and reveals the anatomical position of channels on the brain surface in both adults and children.^43^^–^^46^

### 2.6 Data Analysis

As we found statistically significant differences in mean IQ scores between the ASD and TD groups, which could potentially affect results, we initially attempted to perform an analysis of covariance (ANCOVA) of both the behavioral performance and fNIRS data with the IQ score as a covariate. However, because no significant regression was found, ANCOVA was not appropriate to analyze the data. In addition, no significant correlations were observed in both groups between the IQ scores and behavioral measures (ASD: rs<0.18; TD: rs<0.17) or between the IQ scores and differential neural responses (ASD: r=0.10; and TD: r=0.02). We therefore assessed performance using mixed-design repeated-measures analysis of variance (ANOVA).

#### 2.6.1 Behavioral performance

We analyzed reaction time and both commission and omission errors as measures of behavioral performance. For statistical analysis, we performed a two-way mixed-design repeated-measures (ANOVA) with group (TD versus ASD) and gaze direction (direct versus averted) as between- and within-subjects factors, respectively.

#### 2.6.2 fNIRS data

We used the oxy-Hb signal because of its higher sensitivity to changes in cerebral blood flow,^47^^–^^49^ signal-to-noise ratio,^49^ and retest reliability^50^ compared to those of the deoxy-Hb and total-Hb signals. The raw oxy-Hb concentration data were bandpass-filtered from 0.01 to 0.5 Hz to remove baseline drift and heartbeat pulsations, similar to previous studies.^14^^–^^19^ Blocks with marked motion-related artifacts were removed, and we analyzed the data of the participants for whom more than four out of the six blocks remained in each direct or averted gaze condition. We averaged the waveforms of the oxy-Hb concentration from 4 to 27 s after the go/no-go block onset as the target period. This average was compared to the average of the baseline period from −10 to 0 s before the go block onset, as in the previous studies.^14^^–^^19^

Because we used experimental procedures for measuring neural activity related to a go/no-go task identical to those used in our previous studies,^14^^–^^17^ we selected and analyzed channel 32 (Ch32) as a region of interest (ROI). This channel was located in the region of the right middle frontal gyrus and inferior frontal gyrus (Table 2), where obvious hemodynamic responses related to inhibitory control have been observed in TD individuals.^51^^–^^53^ Moreover, hemodynamic responses within this channel are influenced by social stimuli such as gaze direction.^27^^,^^28^^,^^37^^,^^38^^,^^54^^,^^55^ We averaged the waveforms of the oxy-Hb concentration and evaluated the hemodynamic responses during the task as the difference in averaged z-transformed oxy-Hb concentration for the baseline period (go task with closed eyes) and target period (go/no-go task with direct or averted gaze), as in the previous studies.^56^^,^^57^ The raw data were transformed into z-scores based on the baseline period from 0 to 10 s after the go block onset, and thus could be compared as normalized data.

**Table 2:** Spatial profiles of the target channels.

MNI coordinates						
Ch	x, y, z (SD)	Macroanatomy	Probability (%)	Brodmann area		Probability (%)
32	42.3, 53.3, 25.3 (13.6)	R middle frontal gyrus	83.1	46	Dorsolateral prefrontal cortex	76.3
		R inferior frontal gyrus	16.9	45	pars triangularis Broca’s area	20.8
				10	Frontopolar area	0.0

Note: MNI, Montreal Neurological Institute; R, right.

A two-way ANOVA was conducted for the statistical analysis of oxy-Hb signals at Ch32. Group was used as a between-subjects factor (TD versus ASD), and gaze direction (direct versus averted) as a within-subjects factor.

### 2.7 Correlation Analysis

To evaluate the relationships between individual differences in hemodynamic responses and autistic traits, we examined the correlation between the differential task-related oxy-Hb signal, which is the averaged oxy-Hb signal in the direct gaze condition subtracted from that in the averted gaze condition, and the AQ score with Pearson’s correlation analysis.

## 3 Results

### 3.1 Behavioral Performance

For the reaction times (Table 3), we did not find any significant main effects (Fs<3.18, all ps>0.08). The main effect of gaze direction on commission errors was significant [F (1,44)=5.03, p<0.05, ${\eta_{p}}^{2}=0.10$]. This finding indicates that the commission error rate in the direct gaze condition was significantly higher than that in the averted gaze condition in both groups. However, the main effect of group and the interaction of group and gaze direction were not significant (Fs<1.86, all ps>0.17). No significant effects were observed in the analysis of on omission errors (Fs<3.41, all ps>0.07).

**Table 3:** Behavioral data for ASD and TD subjects.

(a) Means and standard deviations of reaction times and error rates				
Gaze	ASD (n=22)		TD (n=24)	
	Direct	Averted	Direct	Averted
Reaction time (ms) (SD)	414.5 (53.1)	414.2 (56.0)	409.2 (27.1)	416.9 (40.8)
Omission errors (%) (SD)	1.26 (2.48)	0.89 (1.48)	0.58 (0.89)	0.29 (0.90)
Commission errors (%) (SD)	4.61 (4.63)	3.28 (4.48)	2.84 (3.07)	2.26 (2.15)
(b) Two-way ANOVA for gaze direction and group				
	Source	df	F	p
Reaction time	Gaze direction (direct versus averted)	1, 44	2.676	0.109
	Group (ASD versus TD)	1, 44	0.008	0.928
Omission error	Gaze direction (direct versus averted)	1, 44	3.405	0.072
	Group (ASD versus TD)	1, 44	2.193	0.146
Commission error	Gaze direction (direct versus averted)	1, 44	5.029	0.030*
	Group (ASD versus TD)	1, 44	1.851	0.180

Note: ASD, autism spectrum disorder; TD, typical development; Df, degrees of freedom; F, F value; p, p value.

* p<0.05.

### 3.2 fNIRS Analyses

**Fig. 3:**
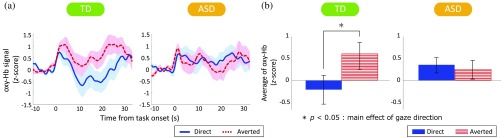
(a) Waveforms of the oxy-hemoglobin (oxy-Hb) signal and (b) comparison of the average oxy-Hb levels in Ch32 between TD children and children with ASD. (a) The waveforms indicate averaged oxy-Hb signals in the direct gaze condition (blue line) and averted gaze condition (red line) for Ch32 located on the right PFC in the TD and ASD groups. (b) Mean oxy-Hb levels from 4 to 24 s for each channel. The blue bars indicate the direct gaze condition, and the red bars indicate the averted gaze condition. The error bars indicate the standard error of the mean.

We found a significant two-way interaction between group and gaze direction for the z-transformed mean oxy-Hb signals [F (1,44)=5.23, p<0.05, ${\eta_{p}}^{2}=0.11$]. To explore the nature of this interaction, tests of simple main effects were performed. The main effect of gaze direction was significant in the TD group [F (1,44)=7.02, p<0.05, ${\eta_{p}}^{2}=0.14$], but not in the ASD group [F (1,44)=0.34, p=0.56, ${\eta_{p}}^{2}=0.01$] (Fig. 3). This result indicates that the oxy-Hb signal in the averted gaze condition was significantly higher than that in the direct gaze condition in the TD group. However, the main effect of group within each gaze direction was not significant [Fs<2.50, all ps>0.11].

### 3.3 Oxy-Hb Signal Data

The time courses of the grand-averaged oxy-Hb signal in Ch32 for the TD and ASD groups are shown in Fig. 3(a). The averaged signals were elevated at the onset of the task in both gaze conditions in both groups; however, they decreased substantially only in the direct gaze condition in the TD group. Therefore, the averaged oxy-Hb signals for the target period had opposite patterns in the two gaze directions in the TD group [Fig 3(b)].

### 3.4 Analysis of Correlations Between Behavioral/Neural Performance and Scores

**Fig. 4:**
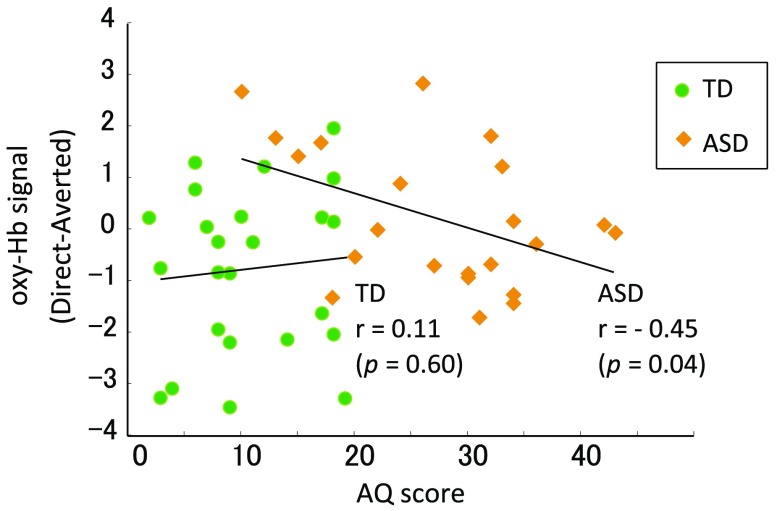
Correlation between oxy-Hb signal in Ch32 (direct minus averted) and AQ score. The scatterplots illustrate how AQ scores were associated with mean differential oxy-Hb signal responses (averaged oxy-Hb signal in the direct gaze condition minus that in the averted gaze condition). The green circles indicate TD participants, and the yellow rhombs indicate participants with ASD. A significant correlation between the oxy-Hb levels and AQ scores was observed only in the ASD group.

We further explored the relationship between the differential task-related oxy-Hb signals (direct versus averted) during the task and behavioral scores. We found a significant negative correlation between the AQ scores and the differential oxy-Hb signals at Ch32 in the ASD group (r=−0.45, p=0.04), but not in the TD group (r=0.11, p=0.60) (Fig. 4). A marginally significant difference was identified between these two correlation coefficients (z=1.88, p=0.06).

## 4 Discussion

In the present study, direct gaze had a significant overall impact on the behavioral data in both groups. There were no significant differences between the ASD and TD groups in reaction time or error rates. The frequency of commission errors in the direct gaze condition was significantly higher than that in the averted gaze condition in both groups. Contrary to the behavioral data, averted gaze had a significant impact on the oxy-Hb responses in the right PFC region in the TD group, but not in the ASD group. The mean oxy-Hb levels were significantly correlated with the AQ scores in the ASD group. These results indicate that gaze direction during the inhibitory task affected behavioral performance in both groups, whereas inhibition-related neural responses in the right PFC region were modulated by gaze direction only in TD individuals. We initially predicted that direct gaze would have a differential impact on both behavioral and neural responses. Our prediction was partly borne out by the mean oxy-Hb response levels, but not by the behavioral measures.

Consistent with previous findings of the significant effect of direct gaze in TD children and adults,^58^^–^^63^ the commission error rate in the direct gaze condition was significantly higher than that in the averted gaze condition in the TD group. However, we found a similar effect in the ASD group. Most previous studies on gaze processing in ASD have reported atypical eye-gaze processing in infancy and childhood.^37^^,^^64^^–^^67^ Furthermore, gaze stimuli have a great impact on other cognitive functions such as memory and face recognition.^38^^,^^55^ These studies have shown that cognitive abilities in TD children are facilitated by direct gaze; however, this is not the case for children with ASD.

Our behavioral findings in the ASD group are inconsistent with the previous results, which may be explained by our task design and instructions. Kikuchi et al.^68^ reported that atypical disengagement from faces was diminished in children with ASD when they were instructed to fixate on the eye region. In line with these findings, when individuals with ASD were instructed to fixate on the eyes, it also altered the hemodynamic responses in the fusiform region and amygdala, which are involved in face processing.^62^^,^^69^ These results imply that the explicit instruction of fixating on the eye region in our experiment may have altered behavioral responses in children with ASD, which in turn may have led to their behavioral performance being comparable to that of the control group.

We found that commission errors in the direct gaze condition were significantly increased compared to those in the averted gaze condition. Previous studies have demonstrated that perceived social stimuli, such as direct gaze, influence various aspects of cognitive performance. For example, when participants fixate on direct-gaze faces, but not on averted-gaze faces, the reaction time is prolonged to detect peripheral targets.^61^^,^^70^^,^^71^ Moreover, a go/no-go task with emotional faces induced a higher rate of commission errors compared to that in a task with nonsocial stimuli.^72^ These findings suggest that social stimuli can capture visual attention. Another possibility is that the significantly increased error rate may be attributable to an elevated arousal level.^73^ Therefore, inhibitory responses may be less functional in the direct gaze condition compared to the averted gaze condition.

In contrast with the behavioral findings, we found differences in the mean oxy-Hb signals at Ch32 depending on the displayed direction of gaze. Ch32 was located in the ROI encompassing the right middle and inferior frontal gyri (Table 2). Although recent neuroimaging studies have demonstrated that the right inferior frontal cortex plays an important role in inhibition control,^10^^,^^74^ meta-analyses have shown that the right-lateral prefrontal cortex, including the middle and inferior frontal gyri, is associated with performance in go/no-go tasks.^53^^,^^75^ Moreover, other studies have found developmental changes in the neural activation pattern during inhibitory tasks: from childhood to adulthood, the pattern shifts from the middle to the inferior frontal gyrus region.^76^^,^^77^ Therefore, given that the participants in the present study were mainly children and adolescents, we consider our ROI selection reasonable. In addition, in our previous fNIRS studies,^14^^–^^19^ we selected Ch32 as an ROI located in the inferior frontal gyrus (22% to 33%) and middle frontal gyrus (63% to 78%), and found robust neural responses during an inhibition task. The mean oxy-Hb signals during the task were significantly more enhanced in the averted gaze condition than in the direct gaze condition in the TD group, but not in the ASD group. Therefore, the right PFC region may be sensitive to social stimuli as part of the social brain network.^78^^,^^79^ Supporting this possibility, several neuroimaging studies on gaze perception have reported that hemodynamic responses in the DLPFC region are also modulated by the perception of gaze direction.^27^^,^^28^^,^^80^ Moreover, the neural responses to gaze direction were modulated differently in the TD and ASD groups.^28^^,^^80^

As we did not find any group differences in behavioral responses, we speculate that differential neural mechanisms may be involved in the prefrontal regions. Several studies have reported that the behavioral performance of ASD individuals is comparable to that of typical controls despite differences in neural activity related to the cognitive task.^81^^–^^84^ In these studies, differential neural responses were observed in the ASD and TD groups during a selective attention task and in socially relevant tasks. In line with the results of these studies, it is likely that atypical gaze information processing (possibly in other brain regions such as the posterior superior temporal sulcus) may have affected neural activity in the PFC region in the ASD group in the present study. Consequently, we observed different neural responses to gaze direction in TD and ASD subjects.

We found a significant positive correlation between the mean oxy-Hb levels and the total AQ scores only in the ASD group. Concordant with these findings, some studies have shown that neural activity and behavioral performance are correlated with autistic traits in ASD.^40^^,^^85^^–^^87^ Two of these studies further analyzed the correlation with autistic traits in a TD group. One reported no correlation,^85^ and the other reported an inverse correlation.^86^ The variable results may be attributable to ASD encompassing a wide continuum of clinical features; however, the mechanism of the differences in correlation is unclear. Further studies are needed to explain the discrepancy found in the present study.

Several limitations should be acknowledged. First, the overall sample size of the present study was small, and the numbers of ASD and TD individuals were different, resulting in a significant, if only marginally, sex difference between the two groups. To increase accuracy, future studies with increased sample sizes are necessary. Second, our study did not examine neural activity in brain regions other than the right PFC. We did not elucidate the involvement of other social brain network regions, most importantly the posterior part of the superior temporal sulcus, which has been implicated in gaze processing by numerous studies.^88^ In addition, recent studies have reported that the PFC is not functionally unique in its inhibitory control.^89^^,^^90^ Further study is needed to investigate how other brain regions related to inhibition and the processing of socially related information may be involved in the task used in the present study. Third, we adopted a block design with 50% of go/no-go trials, similar to other studies using 50% of go/no-go trials^6^^,^^33^^–^^36^ and a block design.^33^^,^^36^^,^^91^^–^^94^ Our previous studies that employed 50% of go/no-go trials revealed robust neural responses in the right PFC.^14^^–^^16^ However, several other studies have shown that a lower fraction of no-go trials, such as 20%, requires an inhibition function called a prepotent response, which in turn induces a stronger overall inhibitory response.^89^^,^^95^ Furthermore, although a block design can have statistical power superior to an event-related design, it cannot easily distinguish responses to specific behavioral outcomes within a block (e.g., correct and incorrect tasks). Therefore, further studies should adopt a task design that induces prepotent responses and an event-related overall design to more clearly reveal the inhibitory responses.

In conclusion, our results suggest that gaze direction has a significant impact on inhibitory control in both behavioral and hemodynamic responses that differs between ASD and TD individuals. Whereas the behavioral measures showed similar patterns in the ASD and TD groups, hemodynamic responses in the right PFC region were modulated differentially by gaze direction in the two groups. These results may reflect the atypical gaze processing mechanisms in the prefrontal regions of ASD individuals during an inhibitory task.
